# Enhanced radiosensitivity in experimental tumours following erythropoietin treatment of chemotherapy-induced anaemia.

**DOI:** 10.1038/bjc.1998.572

**Published:** 1998-09

**Authors:** O. Thews, R. Koenig, D. K. Kelleher, J. Kutzner, P. Vaupel

**Affiliations:** Institute of Physiology and Pathophysiology, Johannes Gutenberg-University, Mainz, Germany.

## Abstract

The radiosensitivity of solid tumours in anaemic rats treated with recombinant human erythropoietin (rhEPO, epoetin beta) was studied. Anaemia was induced by a single dose of carboplatin (45 mg kg(-1) i.v.), resulting in a reduction in the haemoglobin concentration by 30%. In a second group, the development of anaemia was prevented by rhEPO (1000 IU kg(-1)) administered s.c. three times per week starting 6 days before the carboplatin application. Three days after carboplatin treatment, DS-sarcomas were implanted subcutaneously onto the hind foot dorsum. Neither carboplatin nor rhEPO treatment influenced tumour growth rate. Five days after implantation, tumours were irradiated with a single non-curative dose (10 Gy), resulting in a growth delay with a subsequent regrowth of the tumours. In the rhEPO-treated group, the growth delay lasted significantly longer (9.5 days vs. 4.5 days) and the regrowth was slower (6.0 days vs. 4.1 days) compared with the anaemic group. These data suggest that the correction of chemotherapy-induced anaemia by rhEPO (epoetin beta) treatment increases tumour radiosensitivity, presumably as a result of an improved oxygen supply to tumour tissue.


British Journal of Cancer (1998) 78(6), 752–756
© 1998 Cancer Research Campaign

### Enhanced radiosensitivity in experimental tumours following erythropoietin treatment of chemotherapy-induced anaemia

**O Thews[1], R Koenig[1], DK Kelleher[1], J Kutzner[2] and P Vaupel[1]**

[1]Institute of Physiology and Pathophysiology and [2]Department of Radiotherapy, Johannes Gutenberg-University, D-55099 Mainz, Germany

**Summary** The radiosensitivity of solid tumours in anaemic rats treated with recombinant human erythropoietin (rhEPO, epoetin beta) was studied. Anaemia was induced by a single dose of carboplatin (45 mg $kg^{-1}$ i.v.), resulting in a reduction in the haemoglobin concentration by 30%. In a second group, the development of anaemia was prevented by rhEPO (1000 IU $kg^{-1}$) administered s.c. three times per week starting 6 days before the carboplatin application. Three days after carboplatin treatment, DS-sarcomas were implanted subcutaneously onto the hind foot dorsum. Neither carboplatin nor rhEPO treatment influenced tumour growth rate. Five days after implantation, tumours were irradiated with a single non-curative dose (10 Gy), resulting in a growth delay with a subsequent regrowth of the tumours. In the rhEPO-treated group, the growth delay lasted significantly longer (9.5 days vs. 4.5 days) and the regrowth was slower (6.0 days vs. 4.1 days) compared with the anaemic group. These data suggest that the correction of chemotherapy-induced anaemia by rhEPO (epoetin beta) treatment increases tumour radiosensitivity, presumably as a result of an improved oxygen supply to tumour tissue.

**Keywords:** radiosensitivity; anaemia; erythropoietin; epoetin beta; carboplatin

Anaemia is a common phenomenon in clinical oncology which can be caused by the neoplastic disorder itself [due to, for example, deficiency of erythropoietic factors, bone marrow inhibition by inflammatory cytokines, haemolysis, bone marrow infiltration or paraneoplastic syndromes (for a review see Levine et al, 1993; Spivak, 1994)], by myelosuppressive therapy modalities or by acute or chronic bleeding of the tumour. The anaemia can severely affect the general well-being of the patient and may limit the applicability and efficacy of several anti-tumour therapy modalities. Numerous studies have shown a strong relationship between the therapeutic outcome of radiotherapy and haemoglobin concentrations, indicating that anaemic patients have a poorer prognosis following standard radiotherapy [for reviews see (Grau et al, 1998; Levine et al, 1993)].

The diminished therapeutic effect of radiotherapy in anaemic patients might be a result of the reduced oxygen-carrying capacity of the blood, which in turn decreases the arterial oxygen supply to the tumour. Thus, severe anaemia will result in a poorer oxygenation status, further increasing the hypoxia already present in many tumours (Vaupel et al, 1989). In a recent study, Kelleher et al (1996) demonstrated that anaemia leads to a significantly lower median tumour $pO_2$ and a higher fraction of hypoxic $pO_2$ values. At the same time, tumour hypoxia or anoxia protects tumour cells from sparsely ionizing radiation and thus reduces the efficacy of radiotherapy (Bush et al, 1978).

With this in mind, several studies have investigated the effect of correcting anaemia by homologous blood or red blood cell (RBC) transfusion on tumour oxygenation (Kelleher et al, 1995) and the outcome of radiotherapy (Levine et al, 1993; Grau et al, 1998). However, these studies have not been able to show definitive improvements in radiosensitivity when anaemia was corrected, possibly because of the recruitment of patients with already advanced diseases (Levine et al, 1993). Furthermore, blood or RBC transfusion carries a risk of infection transmission and may cause alloimmunization and allergic reactions (Spivak, 1994).

The treatment of anaemic tumour patients with recombinant human erythropoietin (rhEPO) is an alternative to blood transfusion. Besides improving the general well-being of the patient, rhEPO may increase the oxygen-carrying capacity of blood and thus improve tumour oxygenation, as demonstrated by Kelleher et al (1996). However, to date no conclusive studies have been performed investigating the effect of anaemia correction with rhEPO treatment on the radiosensitivity of tumours. In the present study, the effect of preventing a chemotherapy-induced anaemia by rhEPO (epoetin beta) treatment on the radiosensitivity of experimental rat tumours has therefore been studied.

### MATERIALS AND METHODS

### Animals

Male Sprague–Dawley rats (Charles River Wiga, Sulzfeld, Germany; body weight 140–170 g) housed in our animal care facility were used in the study. Animals were allowed access to food and acidified water ad libitum before and throughout the investigation. All experimentation had previously been approved by the regional animal ethics committee and was conducted according to German federal law.

Received 2 December 1997
Revised 16 February 1998
Accepted 27 February 1998

Correspondence to: O Thews, Institute of Physiology and Pathophysiology, University of Mainz, Duesbergweg 6, D-55099 Mainz, Germany

Supported by the Deutsche Krebshilfe (70-1920 Va 2). This study forms part of the doctoral thesis of Ralph Koenig.

### Tumours

Solid DS-sarcomas were induced by injecting DS-sarcoma cells (0.4 ml, approximately $10^4$ cells $\mu l^{-1}$) subcutaneously into the hind food dorsum. Tumours grew as flat, spherical segments and replaced the subcutis and corium completely. Tumour volumes were determined by measuring the three orthogonal diameters of the tumour and using an ellipsoid approximation with the formula $V = d_1 \times d_2 \times d_3 \times \pi/6$. From the volume growth curves, the volume doubling time was calculated during exponential tumour growth.

### Drugs

A prolonged anaemia was induced in all animals by a single i.v. dose of carboplatin (Sigma-Aldrich, Steinheim, Germany; 45 mg $kg^{-1}$ body weight dissolved in isotonic saline at a concentration of 20 mg $ml^{-1}$) into the tail vein 3 days before tumour implantation.

Recombinant human erythropoietin (epoetin beta, Recormon, purity >98%, Boehringer-Mannheim, Mannheim, Germany) was dissolved in isotonic saline and administered (1000 IU $kg^{-1}$) three times per week over 14 days by s.c. injection starting 9 days before tumour implantation. Control animals received equivalent volumes of the solvent. Studies in rats have shown that there is no significant production of antibodies against rhEPO over this treatment period (W Rebel, personal communication).

### Radiation treatment

Tumours were irradiated locally on day 5 after implantation with a single dose of 10 Gy using conventional 100-kV X-rays at a dose rate of 14 Gy $min^{-1}$. The tube was placed 5–10 mm above the tumour and the field was approximately 4 × 4 $cm^2$. Irradiation was carried out under pentobarbital anaesthesia (40 mg $kg^{-1}$ body weight i.p.; Nembutal, Sanofi Ceva, Paris, France) in animals spontaneously breathing room air and placed supine on an isolating polystyrene block to avoid decreases in core temperature.

### Experimental groups

The experimental groups can be summarized as follows:

Group 1 (anaemic, irradiated): animals treated 3 days before tumour implantation with carboplatin and irradiated on day 5 after implantation with a single dose of 10 Gy ($n$ = 8).

Group 2 (non-anaemic, irradiated): animals treated with rhEPO (epoetin beta) three times per week from 9 days before irradiation and up until the day of irradiation. Carboplatin was administered on day –3 and tumours were irradiated on day 5 with a single dose of 10 Gy ($n$ = 9).

Group 3 (anaemic, non-irradiated): animals treated with carboplatin as in group 1 but not irradiated ($n$ = 8).

Group 4 (non-anaemic, non-irradiated): animals treated with rhEPO (epoetin beta) and carboplatin as in group 2 but not irradiated ($n$ = 7).

Data were collected from two identical but temporally independent sets of experiments, whereby all treatment groups were included in each set to ensure reproducibility of the results.

### Measurements

Erythrocyte and leucocyte parameters were assessed using a multiparameter, automated haematology analyser (Cell-Dyn 3500;

**Figure 1** Time course of the haemoglobin concentration (cHb) in animals treated only with a single dose of carboplatin on day –3 (Δ) and animals additionally treated with rhEPO (from day –9 until day +5, three times a week) (●). Day 0 is the day of tumour implantation. Each point represents data from at least eight animals. (**)$P < 0.01$. Arrows indicate the times of rhEPO and carboplatin administration as well as of tumour implantation (TI) and irradiation

**Figure 2** Tumour volume growth in anaemic animals treated with carboplatin (Δ) and animals in which carboplatin-induced anaemia was prevented by preceding treatment with rhEPO (●). Each data point represents a minimum of 12 tumours

Abbott, Wiesbaden, Germany) measuring erythrocyte, white blood cell and platelet count together with the mean cell volume by an impedance technique and the haemoglobin concentration by a photometric method (at 540 nm). In addition, the analyser uses the measured values to calculate several other parameters (e.g. haematocrit, mean corpuscular haemoglobin content and mean corpuscular haemoglobin concentration). All measurements were performed using a sample of venous blood (130 µl) taken from the animal's tail.

### Statistical analysis

Results are expressed as means ± standard error of the mean (s.e.m.). Differences between the groups were assessed by two-tailed Wilcoxon test for unpaired samples. The significance level was set at $\alpha = 5\%$ for all comparisons. For characterizing the effect of radiotherapy on tumour growth, the growth delay induced by irradiation was calculated.

© Cancer Research Campaign 1998 

### RESULTS

Starting at a mean haemoglobin concentration of approximately 130 g $l^{-1}$, a single dose of carboplatin (45 mg $kg^{-1}$ body weight i.v.) resulted in pronounced anaemia in rats with a mean haemoglobin concentration (cHb) of about 90 g $l^{-1}$, 8 days after application. The haemoglobin level remained at this reduced level for at least 7 days (Figure 1) before gradually recovering to normal values over a period of 20 days (data not shown). Continuous treatment with rhEPO (epoetin beta) in otherwise untreated rats increases the cHb within 1 week from 132 g $l^{-1}$ to 149 g $l^{-1}$ (Figure 1). A subsequent application of carboplatin 6 days after commencement of rhEPO therapy reduces the haemoglobin level within 8 days to values comparable to the cHb before rhEPO treatment. Withdrawal of further rhEPO (epoetin beta) application led to the further development of anaemia down to levels of control animals not treated with rhEPO (Figure 1). Thus, rhEPO therapy for 6 days before carboplatin application results in prevention of the chemotherapy-induced anaemia at the time of irradiation.

The volume growth curves in the anaemic control group and the group in which anaemia was prevented by rhEPO treatment were identical, with a volume doubling time of 2.4 ± 0.1 days independent of the actual cHb or treatment with rhEPO for 14 days (Figure 2).

In Figure 3, the tumour growth curve is shown for both groups when tumours were irradiated on day 5 after tumour implantation. Both curves initially show a further volume increase after irradiation, which is then followed by a slight shrinkage of the tumour. After a period of 5–7 days, the tumours began to regrow. On the day of irradiation, RBC-related parameters were significantly different between the two treatment groups (Table 1), with the group treated only with carboplatin showing a mean haemoglobin concentration of 90 g $l^{-1}$ and the group in which anaemia was prevented by previous rhEPO (epoetin beta) treatment showing a cHb of 127 g $l^{-1}$ (comparable to the cHb of the pretreatment period). In both groups, the parameters describing characteristics of single erythrocytes (mean cell volume, mean corpuscular haemoglobin content and mean corpuscular haemoglobin concentration) showed no difference and are within the normal range, indicating a normocytic, normochromic anaemia induced by carboplatin (Table 1). After irradiation, the tumour growth rate was significantly different between the groups (Figure 3), with tumours in anaemic animals regrowing faster than in non-anaemic animals (volume doubling time during the regrowth period was 4.1 days in the anaemic group, with 1 out of 12 tumours showing regression after irradiation; and 6.0 days in the non-anaemic, rhEPO-treated group with 4 out of 18 tumours showing regression). The regrowth after irradiation also starts later in the epoetin beta group, resulting in a growth delay (at the 1.3 ml tumour volume level) of approximately 9.5 days compared with 4.5 days in the anaemic group and 12.0 days in non-anaemic control animals (data not shown).

**Table 1** RBC-related parameters on the day of irradiation in the anaemic carboplatin group and the carboplatin group in which anaemia was prevented by rhEPO (epoetin beta) treatment. Additionally, for comparison, values in non-anaemic, untreated animals are given. ($n$ = number of animals; $P$-values for the comparison of anaemic control vs. rhEPO group)

| | Carboplatin-treated | Carboplatin +rhEPO-treated | Untreated controls |
|---|---|---|---|
| $n$ | 8 | 9 | 7 |
| cHb (g $l^{-1}$) | 90 ± 8 | 127 ± 5 | 137 ± 7 |
| | $P$ = 0.0017 | | |
| Haematocrit (%) | 31 ± 3 | 45 ± 1 | 42 ± 1 |
| | $P$ = 0.0009 | | |
| RBC count ($10^6$ $\mu l^{-1}$) | 4.7 ± 0.4 | 6.5 ± 0.2 | 7.0 ± 0.2 |
| | $P$ = 0.0009 | | |
| Mean corpuscular volume (MCV) (fl) | 66 ± 1 | 69 ± 2 | 60 ± 1 |
| | n.s. | | |
| Mean corpuscular haemoglobin (MCH) (pg) | 19 ± 1 | 20 ± 1 | 20 ± 1 |
| | n.s. | | |
| Mean corpuscular cHb (MCHC) (g $l^{-1}$) | 287 ± 5 | 284 ± 5 | 330 ± 2 |
| | n.s. | | |

**Figure 3** Tumour volume growth in animals with carboplatin-induced anaemia (Δ) and animals in which anaemia was prevented by preceding treatment with rhEPO (●). All tumours were irradiated with a single dose of 10 Gy on day 5. Each data point represents a minimum of 12 tumours. Arrows indicate the times of rhEPO administration and irradiation. *$P$ < 0.05, **$P$ < 0.01

### DISCUSSION

Carboplatin at an i.v. dose of 45 mg $kg^{-1}$ body weight induces a pronounced and prolonged normochromic, normocytic anaemia. At this dose, the mean haemoglobin concentration is reduced by about 30% of the control level with a nadir on day 11 (data not shown) and a pronounced variability ranging from 42 to 125 g $l^{-1}$. The dose used is close to the maximally tolerated dose of carboplatin (60 mg $kg^{-1}$ body weight i.v.) (Siddik et al, 1987). The data on carboplatin-induced anaemia obtained in the present study are in good accordance with observations made by other investigators (Siddik et al, 1987; Ohno et al, 1993), describing a dose-dependent induction of anaemia with carboplatin doses between 40 and 60 mg $kg^{-1}$. Although Siddik et al (1987) concluded that internal haemorrhaging as a result of thrombocytopenia causes the carboplatin-induced anaemia, no signs of severe bleeding were observed in the present study. Similarly, carboplatin-induced anaemia has also been attributed to myelosuppression. However, rhEPO treatment has been shown to be effective in increasing the haemoglobin concentration in these carboplatin-treated patients (Markman et al, 1993; de Campos et al, 1995). In the present study, rhEPO was able to prevent anaemia if it was administered before carboplatin. However, if carboplatin was given first and anaemia therefore already present, rhEPO treatment could only slightly improve the RBC-related parameters (data not shown).

© Cancer Research Campaign 1998

As many chemotherapeutic agents are myelosuppressive, the anaemia model used in the present study describes a realistic situation of patients undergoing a combined radiochemotherapy. The model of a tumour-associated anaemia developed earlier by our group (Kelleher et al, 1996) could not be applied to the present study, as the ascites tumour used to induce anaemia cannot be used for an observation period greater than 6–8 days.

The carboplatin treatment 3 days before tumour implantation had no effect on the growth rate of the DS-sarcoma. The volume doubling time of 2.4 days found in the non-irradiated group is in good accordance with previous data obtained for this tumour model (Busse et al, 1995). As the biological half-life of carboplatin is about 3–4 h in humans (Reece et al, 1987), and the turnover is somewhat higher in rats, it can be assumed that on the day of tumour implantation no appreciable amounts of carboplatin are present in the animals, and therefore effects of carboplatin on tumour growth in this model are not to be expected.

As the growth curves of the non-irradiated rhEPO and control group are not different, it can be concluded that rhEPO per se has no effect on the growth rate of tumours. Joiner et al (1993) found in an in vivo study a slight slowing of tumour growth rate in the first days following implantation in a mouse model treated with higher rhEPO doses. Thereafter, tumour growth rate did not differ between the groups.

Tumour growth in chronically hypoxic animals is slower (Tannock et al, 1970). The significantly reduced tumour growth rate in anaemic animals observed by McCormack et al (1990) has, therefore, been attributed to increases in tumour hypoxia. In the present study, no evidence was found for differences in tumour growth between anaemic animals and rhEPO-treated anaemic animals, as was the case in our earlier study with a tumour-induced anaemia (Kelleher et al, 1996). The differences between the findings of McCormack's and our group cannot be explained. Factors such as tumour blood flow and blood rheology may play a role and warrant further investigation.

Several studies have been performed to analyse the effect of anaemia on the radiosensitivity of solid tumours. However, the results of these studies are not unanimous, describing an increase in radioresistance (Hewitt et al, 1971; Hill et al, 1972; Hirst et al, 1984; McCormack et al, 1990), no effect on the outcome of radiotherapy (Hirst et al, 1984; Joiner et al, 1993) but also an increase in radiosensitivity (Rojas et al, 1987). One major factor affecting radioresistance during anaemia seems to be the period of time over which anaemia occurred. Pronounced differences were seen between studies in which anaemia was acutely (Hewitt et al, 1971; Hirst et al, 1984) or chronically (Hirst et al, 1984; Rojas et al, 1987; Joiner et al, 1993) induced. From these data, it seems obvious that physiological adaptation to haematocrit changes plays a role in the effect of anaemia on radiosensitivity. Another factor influencing the results might be the application of irradiation as a single dose or in a fractionated schedule (Hirst et al, 1984; Rojas et al, 1987).

On the basis of these findings, several studies have investigated the effect of correcting anaemia on sensitivity to radiotherapy (Hewitt et al, 1971; Hill et al, 1972; Hirst et al, 1984; Rojas et al, 1987; Joiner et al, 1993), whereby in most of these studies blood or RBC transfusion was used to compensate for anaemia. Studies on the effects of transfusion on radioresistance have generally shown an improvement in radiotherapy with increases in haemoglobin concentration (Hewitt et al, 1971; Hirst et al, 1984; Rojas et al, 1987). This effect was even seen in a study in which anaemia initially increased the radiosensitivity (Rojas et al, 1987).

Most studies of the effect of rhEPO on the anaemic state during radiotherapy have generally only focused on changes in RBC-related parameters (Lavey et al, 1993; Vijayakumar et al, 1993) and did not examine changes in radiosensitivity. Only Joiner et al (1993) used rhEPO to correct a tumour-associated anaemia and measured radiosensitivity in anaemic animals as well as in mice in which anaemia was treated with different doses of rhEPO. However, the anaemia model Joiner et al used (carcinoma NT in CBA mice) results in only moderate anaemia with a haematocrit of 38%. The correction of the anaemia by rhEPO (20 U daily) led to a haematocrit of 65%, an overcompensation which can be interpreted as a rhEPO-induced polycythaemia. It is questionable whether a haematocrit of 38% would result in a reduction in the oxygen transport capacity, which in turn could appreciably increase tumour hypoxia. At the same time, the rheological properties of blood at a haematocrit of 65% could result in a decrease in tumour perfusion, as seen by Joiner et al, (1993), which might lead to a reduction in oxygen supply to the tumour. Thus the overcompensation of anaemia in that study could be the reason for a lack of improvement in radiosensitivity. In the present study, the anaemia group showed a haematocrit of 31% and after correction by rhEPO of 45%. At these levels, anaemia has a measurable impact on the oxygenation of the DS-sarcoma (Kelleher et al, 1996). Here, the fraction of $pO_2$ values between 0 and 2.5 mmHg (indicating less than half-maximum radiosensitivity) was 76 ± 3% for anaemic animals and 55 ± 3% for animals in which anaemia was prevented by rhEPO treatment, at a tumour volume comparable to that used in this study on the day of irradiation. These differences ($P < 0.001$) in the fraction of hypoxic $pO_2$-values might explain the increase in radiosensitivity seen following rhEPO in this study.

In conclusion, the present study has demonstrated that correction of a clinically relevant anaemia (cHb approximately 90 g $l^{-1}$) by treatment with rhEPO (epoetin beta) can significantly increase the radiosensitivity of solid growing DS-sarcomas, tumours showing pronounced hypoxia even under non-anaemic control conditions (Kelleher et al, 1996). These results form a basis for further studies on improving the outcome of radiotherapy in anaemic patients by rhEPO treatment, especially in patients in whom tumours are known to be hypoxic. In particular, the schedule of rhEPO treatment should be further investigated, for example to assess whether administration of rhEPO after chemotherapy is necessary to obtain the same radiosensitizing effect.

### ACKNOWLEDGEMENTS

The authors wish to thank Boehringer-Mannheim (Mannheim, Germany) for the generous donation of recombinant human erythropoietin (epoetin beta). DS-sarcoma was provided by Dr H Loehrke from the Tumour Bank of the German Cancer Research Centre in Heidelberg.

### REFERENCES

Bush RS, Jenkin RDT, Allt WEC, Beale FA, Dembo AJ and Pringle JF (1978) Definitive evidence for hypoxic cells influencing cure in cancer therapy. *Br J Cancer* **37** (suppl. 3): 302–306

Busse M and Vaupel PW (1995) The role of tumor volume in 'reoxygenation' upon cyclophosphamide treatment. *Acta Oncol* **34**: 405–408

de Campos E, Radford J, Steward W, Milroy R, Dougal M, Swindell R, Testa N and Thatcher N (1995) Clinical and in vitro effects of recombinant human erythropoietin in patients receiving intensive chemotherapy for small-cell lung cancer. *J Clin Oncol* **13**: 1623–1631

© Cancer Research Campaign 1998

Grau C and Overgaard J (1998) Significance of hemoglobin concentration for treatment outcome. In *Blood Perfusion and Microenvironment of Human Tumors – Implications for Clinical Radiology*. Molls M and Vaupel P (eds), pp. 101–112. Springer-Verlag: Berlin

Hewitt HB and Blake ER (1971) Effect of induced host anaemia on the viability and radiosensitivity of murine malignant cells in vivo. *Br J Cancer* **25**: 323–336

Hill RP, Bush RS and Yeung P (1972) The effect of anemia on the fraction of hypoxic cells in an experimental tumor. *Br J Radiol* **44**: 299–304

Hirst DG, Hazelhurst JL and Brown JM (1984) The effect of alterations in hematocrit on tumor sensitivity to X-rays. *Int J Radiat Biol* **46**: 345–354

Joiner B, Hirst VK, McKeown SR, McAleer JJA and Hirst DG (1993) The effect of recombinant human erythropoietin treatment on tumour radiosensitivity and cancer-associated anaemia in the mouse. *Br J Cancer* **68**: 720–726

Kelleher DK, Matthiensen U, Thews O and Vaupel P (1995) Tumor oxygenation in anemic rats – effects of erythropoietin treatment versus red blood cell transfusion. *Acta Oncol* **34**: 379–384

Kelleher DK, Matthiensen U, Thews O and Vaupel P (1996) Blood flow, oxygenation, and bioenergetic status of tumors after erythropoietin treatment in normal and anemic rats. *Cancer Res* **56**: 4728–4734

Lavey RS and Dempsey WH (1993) Erythropoietin increases hemoglobin in cancer patients during radiation therapy. *Int J Radiat Oncol Biol Phys* **27**: 1147–1152

Levine EA and Vijayakumar S (1993) Blood transfusion in patients receiving radical radiotherapy: a reappraisal. *Onkologie* **16**: 79–87

Markman M, Reichman B, Hakes T, Rubin S, Jones W, Lewis JL, Barakat R, Curtin J, Almadrones L and Hoskins W (1993) The use of recombinant human erythropoietin to prevent carboplatin-induced anemia. *Gynecol Oncol* **49**: 172–176

McCormack M, Nias AHW and Smith E (1990) Chronic anaemia, hyperbaric oxygen and tumour radiosensitivity. *Br J Radiol* **63**: 752–759

Ohno S, Strebel FR, Stephens LC, Siddik ZH, Baba H, Makino M, Khokhar AR and Bull JMC (1993) Haematological toxicity of carboplatin and cisplatin combined with whole body hyperthermia in rats. *Br J Cancer* **68**: 469–474

Reece PA, Bishop JF, Olver IN, Stafford I, Hillcoat BL and Morstyn G (1987) Pharmacokinetics of unchanged carboplatin (CBDCA) in patients with small cell lung carcinoma. *Cancer Chemother Pharmacol* **19**: 326–330

Rojas A, Stewart FA, Smith KA, Soranson JA, Randhawa VS, Stratford MRL and Denekamp J (1987) Effect of anemia on tumor radiosensitivity under normo and hyperbaric conditions. *Int J Radiat Oncol Biol Phys* **13**: 1681–1689

Siddik ZH, Boxall FE and Harrap KR (1987) Haematological toxicity of carboplatin in rats. *Br J Cancer* **55**: 375–379

Spivak JL (1994) Recombinant human erythropoietin and the anemia of cancer. *Blood* **84**: 997–1004

Tannock IF and Steel GG (1970) Tumor growth and cell kinetics in chronically hypoxic animals. *J Natl Cancer Inst* **45**: 123–133

Vaupel P, Kallinowski F and Okunieff P (1989) Blood flow, oxygen and nutrient supply, and metabolic microenvironment of human tumors: a review. *Cancer Res* **49**: 6449–6465

Vijayakumar S, Roach M, Wara W, Chan SK, Ewing C, Rubin S, Sutton H, Halpern H, Awan A, Houghton A, Quiet C and Weichselbaum R (1993) Effect of subcutaneous recombinant human erythropoietin in cancer patients receiving radiotherapy: preliminary results of a randomized, open-labeled, phase II trial. *Int J Radiat Oncol Biol* **26**: 721–729

*© Cancer Research Campaign 1998*


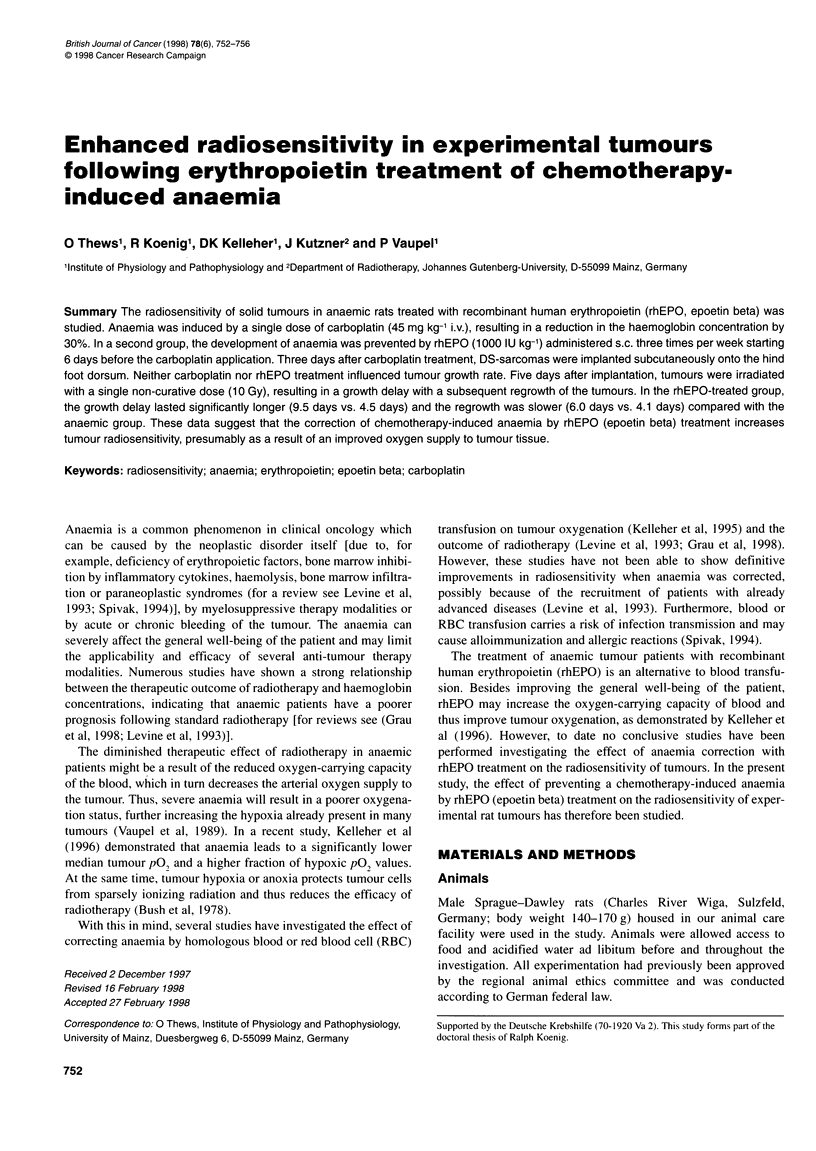

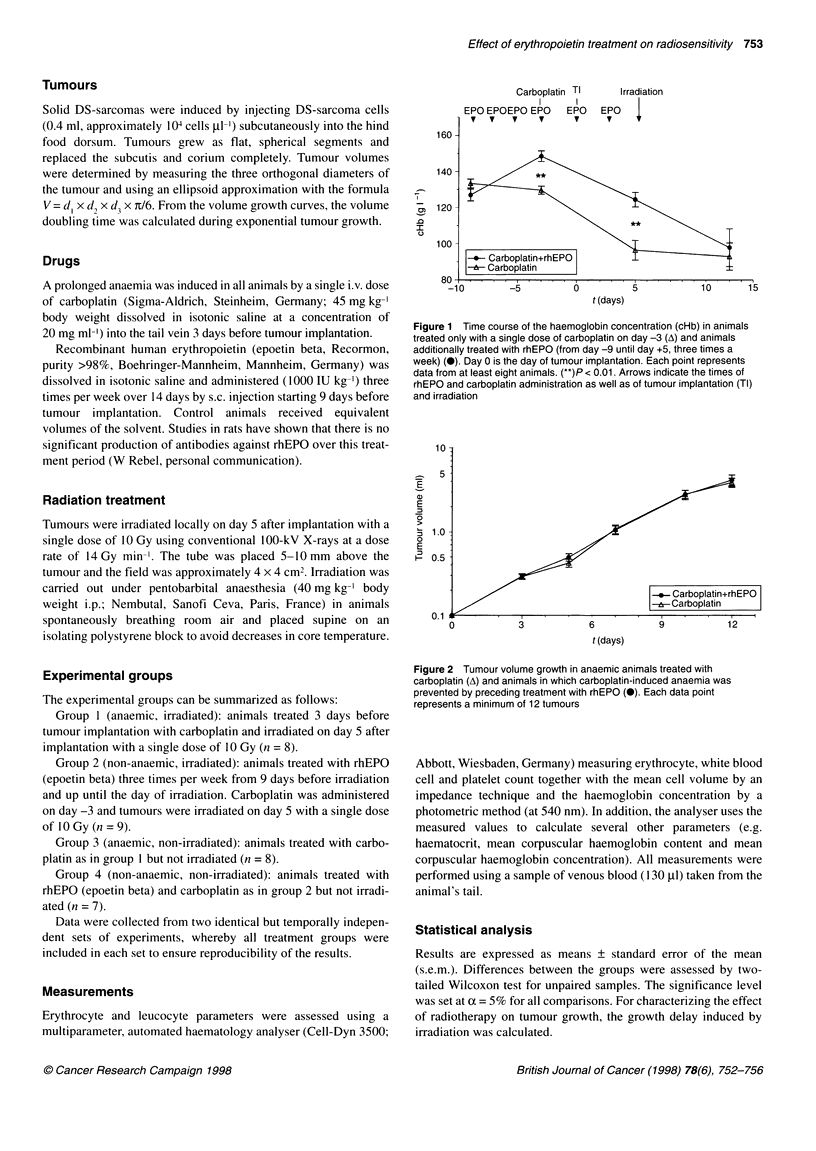

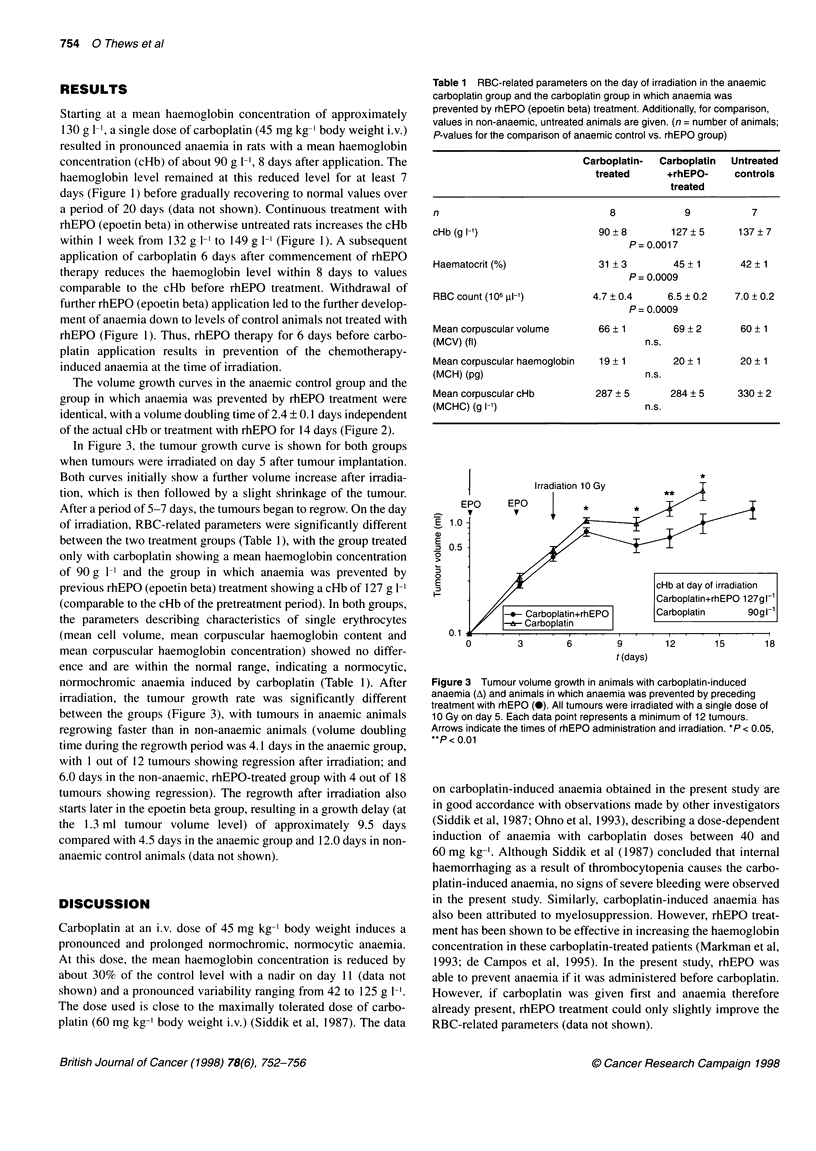

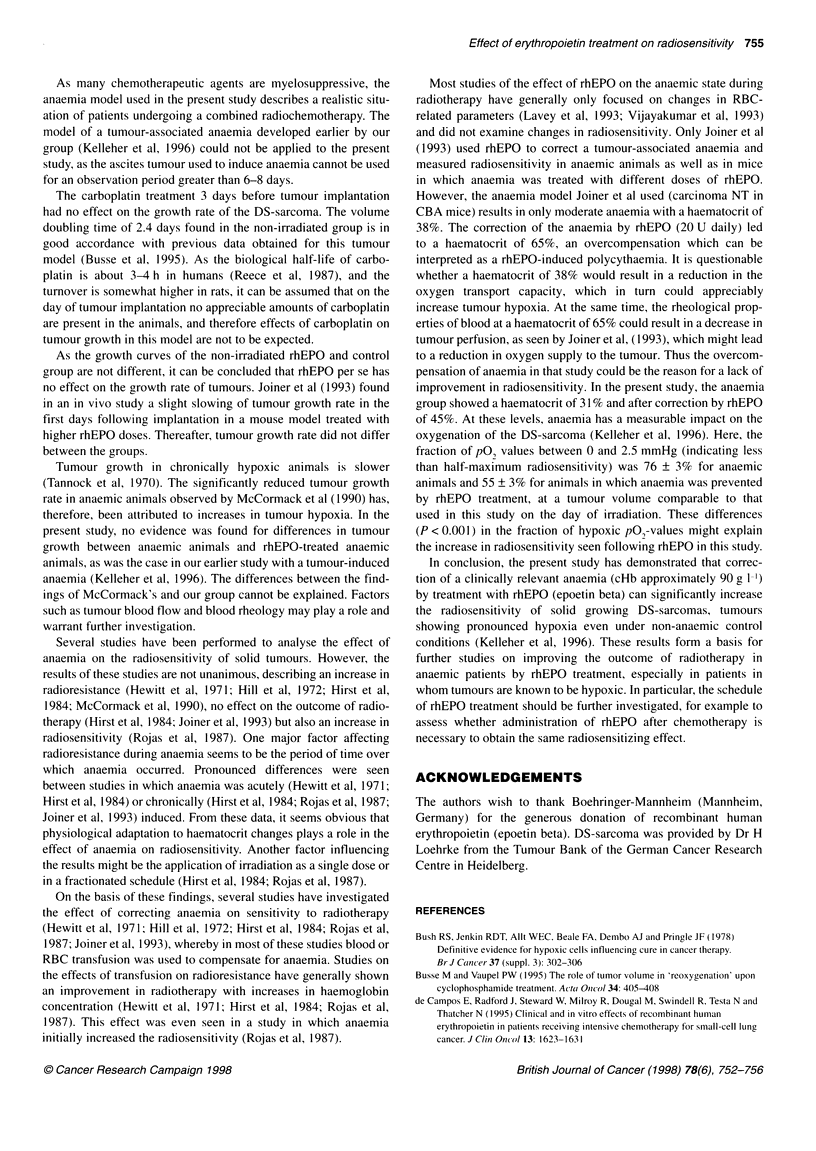

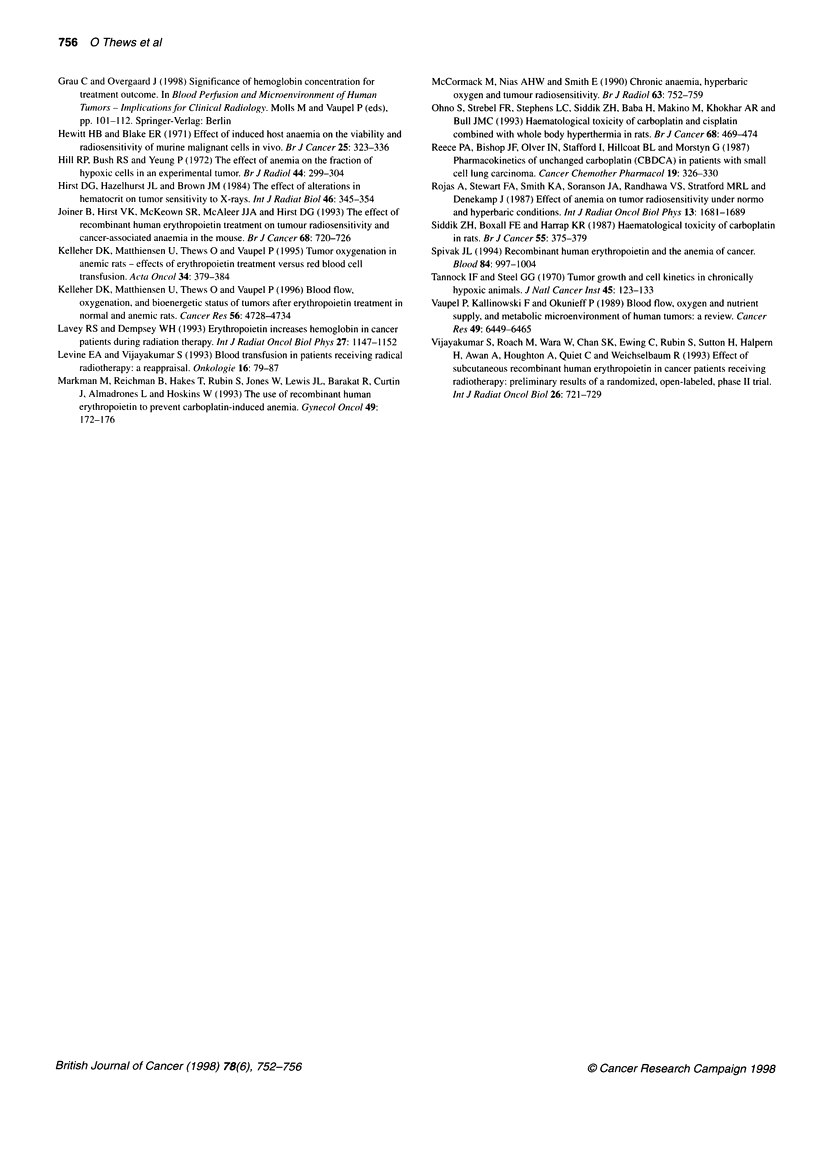


## References

[OCR_00552] Bush R. S., Jenkin R. D., Allt W. E., Beale F. A., Bean H., Dembo A. J., Pringle J. F. (1978). Definitive evidence for hypoxic cells influencing cure in cancer therapy.. Br J Cancer Suppl.

[OCR_00559] Busse M., Vaupel P. W. (1995). The role of tumor volume in 'reoxygenation' upon cyclophosphamide treatment.. Acta Oncol.

[OCR_00579] Hewitt H. B., Blake E. R. (1971). Effect of induced host anaemia on the viability and radiosensitivity of murine malignant cells in vivo.. Br J Cancer.

[OCR_00582] Hill R. P., Bush R. S., Yeung P. (1971). The effect of anaemia on the fraction of hypoxic cells in an experimental tumour.. Br J Radiol.

[OCR_00586] Hirst D. G., Hazlehurst J. L., Brown J. M. (1984). The effect of alterations in haematocrit on tumour sensitivity to X-rays.. Int J Radiat Biol Relat Stud Phys Chem Med.

[OCR_00590] Joiner B., Hirst V. K., McKeown S. R., McAleer J. J., Hirst D. G. (1993). The effect of recombinant human erythropoietin treatment on tumour radiosensitivity and cancer-associated anaemia in the mouse.. Br J Cancer.

[OCR_00595] Kelleher D. K., Matthiensen U., Thews O., Vaupel P. (1995). Tumor oxygenation in anemic rats: effects of erythropoietin treatment versus red blood cell transfusion.. Acta Oncol.

[OCR_00605] Lavey R. S., Dempsey W. H. (1993). Erythropoietin increases hemoglobin in cancer patients during radiation therapy.. Int J Radiat Oncol Biol Phys.

[OCR_00613] Markman M., Reichman B., Hakes T., Rubin S., Jones W., Lewis J. L., Barakat R., Curtin J., Almadrones L., Hoskins W. (1993). The use of recombinant human erythropoietin to prevent carboplatin-induced anemia.. Gynecol Oncol.

[OCR_00622] McCormack M., Nias A. H., Smith E. (1990). Chronic anaemia, hyperbaric oxygen and tumour radiosensitivity.. Br J Radiol.

[OCR_00626] Ohno S., Strebel F. R., Stephens L. C., Siddik Z. H., Baba H., Makino M., Khokhar A. R., Bull J. M. (1993). Haematological toxicity of carboplatin and cisplatin combined with whole body hyperthermia in rats.. Br J Cancer.

[OCR_00631] Reece P. A., Bishop J. F., Olver I. N., Stafford I., Hillcoat B. L., Morstyn G. (1987). Pharmacokinetics of unchanged carboplatin (CBDCA) in patients with small cell lung carcinoma.. Cancer Chemother Pharmacol.

[OCR_00636] Rojas A., Stewart F. A., Smith K. A., Soranson J. A., Randhawa V. S., Stratford M. R., Denekamp J. (1987). Effect of anemia on tumor radiosensitivity under normo and hyperbaric conditions.. Int J Radiat Oncol Biol Phys.

[OCR_00641] Siddik Z. H., Boxall F. E., Harrap K. R. (1987). Haematological toxicity of carboplatin in rats.. Br J Cancer.

[OCR_00645] Spivak J. L. (1994). Recombinant human erythropoietin and the anemia of cancer.. Blood.

[OCR_00649] Tannock I. F., Steel G. G. (1970). Tumor growth and cell kinetics in chronically hypoxic animals.. J Natl Cancer Inst.

[OCR_00653] Vaupel P., Kallinowski F., Okunieff P. (1989). Blood flow, oxygen and nutrient supply, and metabolic microenvironment of human tumors: a review.. Cancer Res.

[OCR_00658] Vijayakumar S., Roach M., Wara W., Chan S. K., Ewing C., Rubin S., Sutton H., Halpern H., Awan A., Houghton A. (1993). Effect of subcutaneous recombinant human erythropoietin in cancer patients receiving radiotherapy: preliminary results of a randomized, open-labeled, phase II trial.. Int J Radiat Oncol Biol Phys.

[OCR_00563] de Campos E., Radford J., Steward W., Milroy R., Dougal M., Swindell R., Testa N., Thatcher N. (1995). Clinical and in vitro effects of recombinant human erythropoietin in patients receiving intensive chemotherapy for small-cell lung cancer.. J Clin Oncol.

